# Supplementary material for: Dynamics of alternative splicing during somatic cell reprogramming reveals functions for RNA-binding proteins CPSF3, hnRNP UL1, and TIA1
Source: Genome Biol. 2021 Jun 3;22:171. doi: 10.1186/s13059-021-02372-5 (PMC8173870; doi:10.1186/s13059-021-02372-5)
Supplement: Supplementary file 1 — Additional file 1. Supplementary figures and corresponding legends. [file 13059_2021_2372_MOESM1_ESM.pdf]

Additional File 1 for

Dynamics of alternative splicing during somatic cell reprogramming reveals functions for RNA-binding proteins CPSF3, hnRNP UL1 and TIA1

Claudia Vivori[1,2,§], Panagiotis Papasaikas[1,#], Ralph Stadhouders[1,##], Bruno Di Stefano[1,%], Anna Ribó Rubio[1], Clara Berenguer Balaguer[1,%%], Serena Generoso[1,2], Anna Mallol[1], José Luis Sardina[1,%%], Bernhard Payer[1,2], Thomas Graf[1,2] and Juan Valcárcel[1,2,3*]

* Correspondence to juan.valcarcel@crg.eu

1 Centre for Genomic Regulation (CRG), The Barcelona Institute of Science and Technology, Carrer del Dr. Aiguader 88, 08003 Barcelona, Spain
2 Universitat Pompeu Fabra (UPF), Carrer del Dr. Aiguader 88, 08003 Barcelona, Spain
3 Institució Catalana de Recerca i Estudis Avançats (ICREA), Passeig Lluís Companys 23, 08010 Barcelona, Spain

§ Current address: The Francis Crick Institute, 1 Midland Road, London NW1 1AT, UK

# Current address: Friedrich Miescher Institute for Biomedical Research, Maulbeerstrasse 66 / Swiss Institute of Bioinformatics, 4058 Basel, Switzerland

Current address: Departments of Pulmonary Medicine and Cell Biology, Erasmus MC, Rotterdam, The Netherlands

% Current address: Department of Molecular Biology, Massachusetts General Hospital / Center for Regenerative Medicine / Center for Cancer Research, Massachusetts General Hospital / Harvard Medical School, Boston, MA, USA / Department of Stem Cell and Regenerative Biology / Harvard Stem Cell Institute, Harvard University, Cambridge, MA, USA

%% Current address: Josep Carreras Leukaemia Research Institute, Carretera de Can Ruti, Camí de les Escoles, s/n, 08916 Badalona, Spain

Supplementary Figures

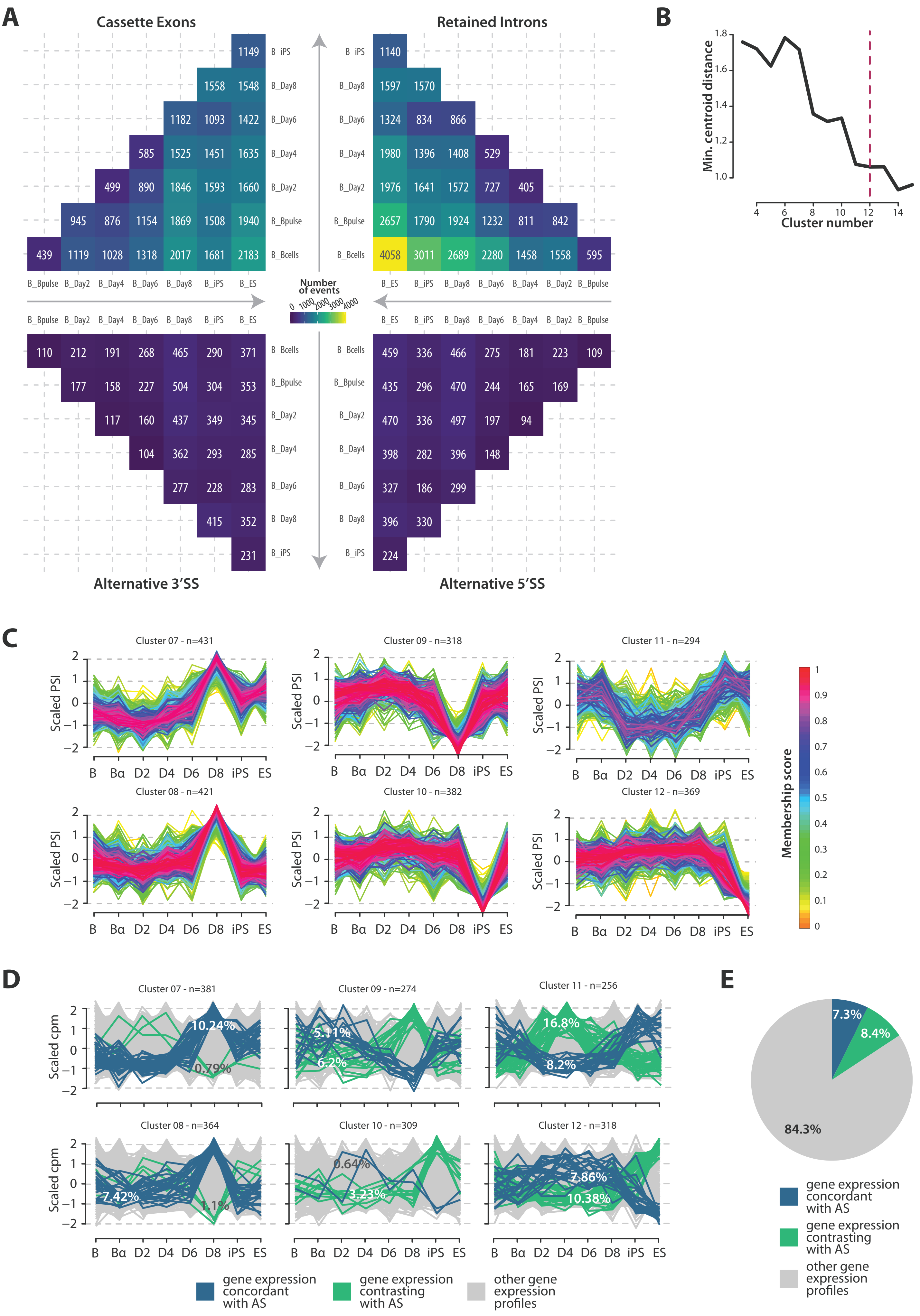

Figure S1

(legend on next page)

Figure S1. (A) Heatmaps representing the number of AS events for each AS category (Cassette exons, retained introns, alternative 3′ and 5′ splice sites) which are differentially spliced between each pair of samples across the C/EBP-mediated reprogramming dataset. The direction of reprogramming time is indicated on the axes by arrows. Related to Figure 1B. **(B)** Variation of the minimum centroid distance score with the number of clusters produced, according to which 12 clusters were selected (magenta dashed line). **(C)** AS clusters not shown in Figure 1C. The size of each cluster is indicated (n). **(D)** Gene expression patterns of genes containing the exons belonging to each of the AS clusters in panel C. Genes with expression correlating with the cluster centroid or its negative (membership > 0.3) are highlighted in blue or green, respectively. Percentage of concordant/contrasting patterns are displayed for each cluster. **(E)** Pie chart indicating the fraction of genes whose gene expression changes are concordant / contrasting with the profiles of AS changes corresponding to their respective cassette exons (average of all the clusters). Colors as in panel G.

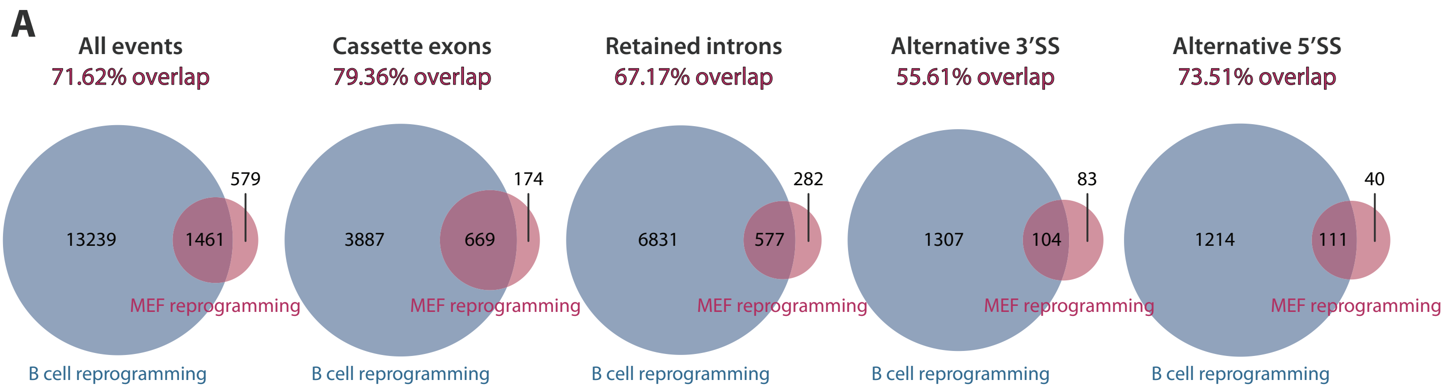

Figure S2. (A) Venn diagrams representing the overlap of events differentially spliced between B cell reprogramming (grey) and MEF reprogramming (magenta) from (1).

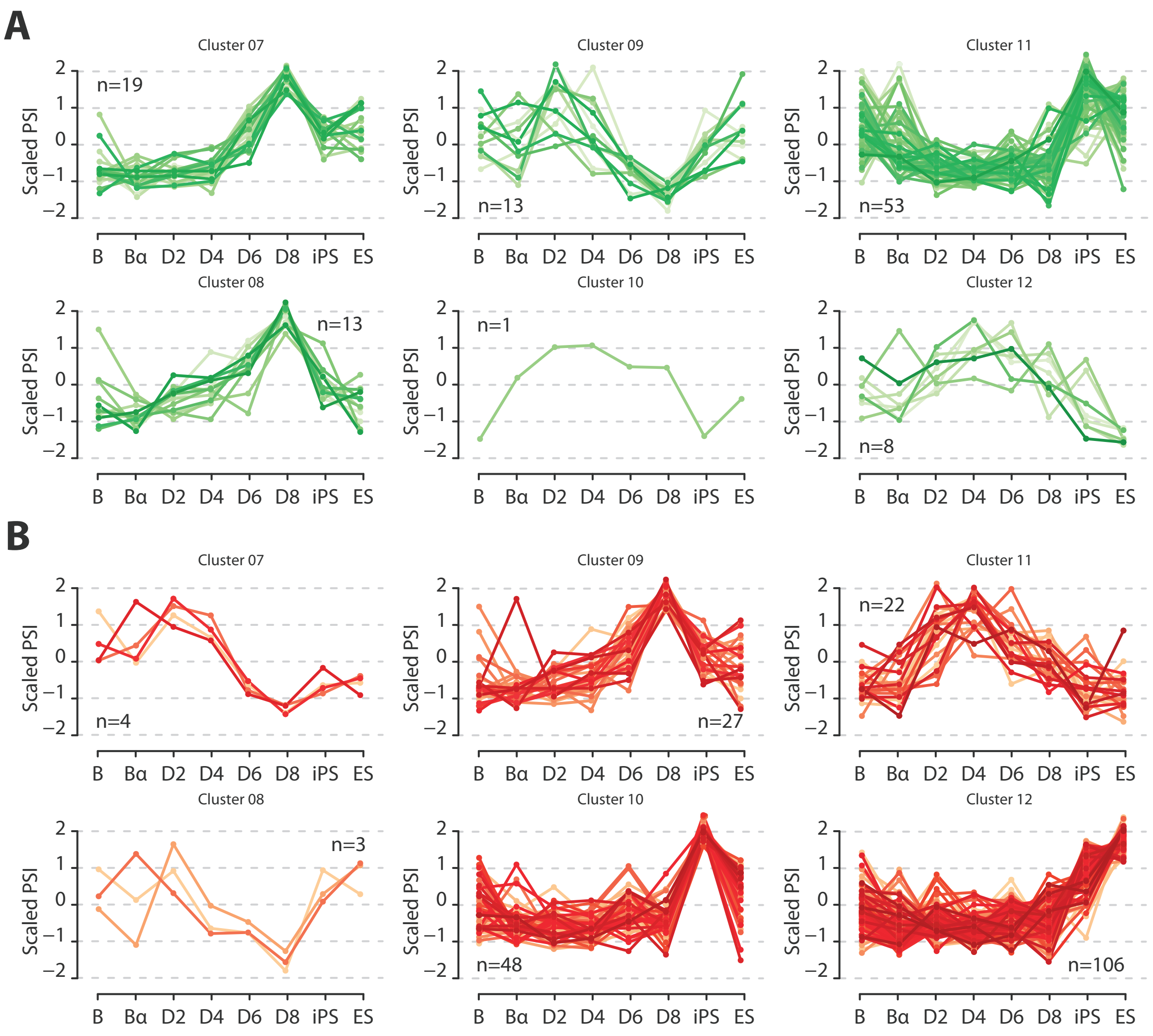

Figure S3. (A) Gene expression profiles of RNA-binding proteins correlating with the centroid of each of the remaining AS clusters (shown in Figure S1C and not in 1C) (positive regulators). Average scaled cpm values are represented by each line and the number of regulators of each cluster is indicated (n). **(B)** Gene expression profiles of RNA-binding proteins correlating with the negative of the centroid of each of the remaining AS clusters (shown in Figure S1C and not in 1C) (negative regulators). Average scaled cpm values are represented by each line and the number of regulators in each cluster is indicated (n).

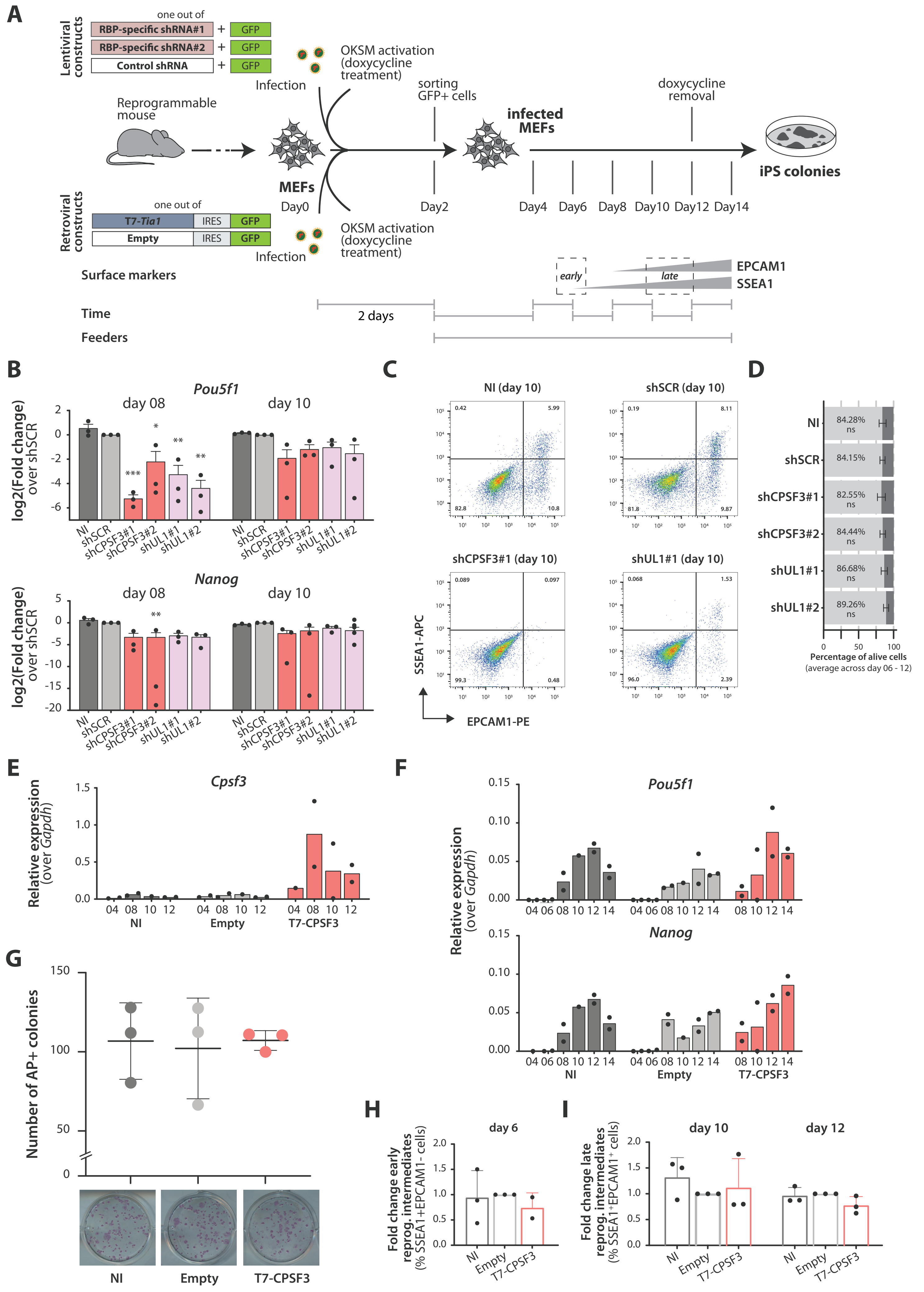

Figure S4

(legend on next page)

Figure S4. (A) Schematic representation of the protocol used to knockdown *Cpsf3* and *Hnrnpul1* or overexpress T7-tagged *Tia1* at early stages of MEF reprogramming. **(B)** Gene expression levels of endogenous *Pou5f1* and *Nanog* mRNAs during MEF reprogramming quantified by RT-qPCR and represented as log2(Fold change) for every condition compared to the shSCR control. Average of biological triplicates and SEM are shown. Statistical significance was calculated by two-way ANOVA on ΔΔCt values comparing to shSCR (*, **, *** = p value < 0.05, 0.01, 0.001 respectively, corrected for multiple testing with Sidak method). **(C)** Examples of gating set ups for cell sorting of SSEA1 and EPCAM1 markers at day 10 of reprogramming. Numbers within the rectangles indicate percentages of cells in each gate. **(D)** Barplot representing the percentage of alive (DAPI$^-$, light gray) and dead (DAPI$^+$, dark gray) cells. Average and SD of days 6, 8, 10 and 12 are shown for each condition. Statistical significance was calculated by Fisher's exact test against shSCR condition (*, **, *** = p value < 0.05, 0.01, 0.001 respectively). **(E)** Expression levels of *Cpsf3* mRNA relative to *Gapdh*, quantified by RT-qPCR in non-infected cells (NI), cells transduced with an empty vector (Empty) or with T7-*Cpsf3* cDNA. **(F)** Expression levels of *Pou5f1* and *Nanog* relative to *Gapdh,* quantified by RT-qPCR as in panel A. **(G)** Number of alkaline phosphatase (AP) positive colonies at day 14 post-OSKM induction upon T7-*Cpsf3* overexpression. Images of representative wells are shown below. **(H)** Percentage of SSEA1+EPCAM1- early reprogramming intermediates (day 6 post-OSKM induction) upon T7-*Cpsf3* overexpression determined by flow cytometry. **(I)** Percentage of SSEA1+EPCAM1+ late reprogramming intermediates (days 10 and 12 post-OSKM induction) upon T7-*Cpsf3* overexpression.

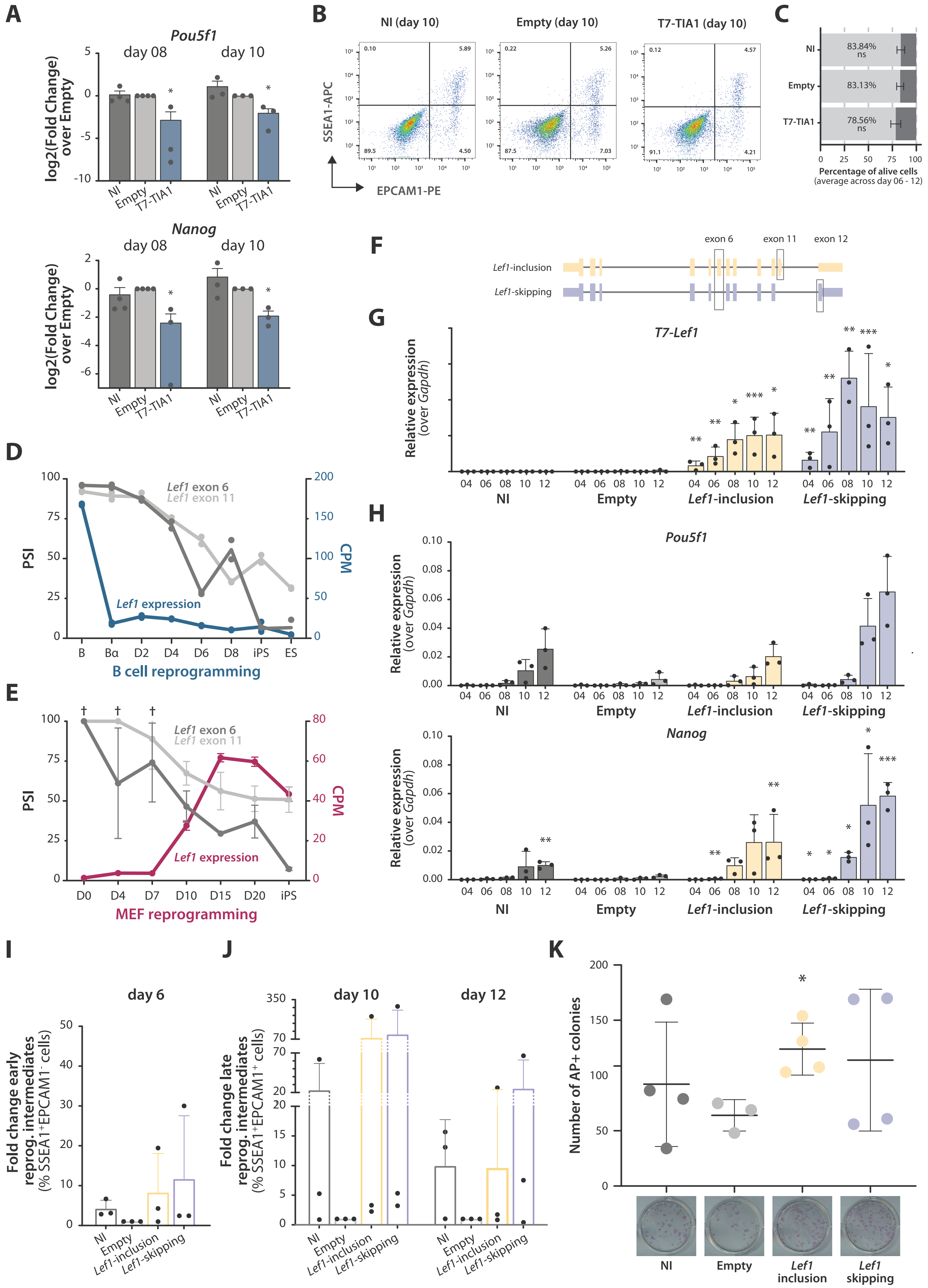

Figure S5

(legend on next page)

Figure S5. (A) Expression levels of endogenous *Pou5f1* and *Nanog* mRNAs during MEF reprogramming upon T7-TIA1 overexpression quantified by RT-qPCR and represented as log2(Fold change) of every condition compared to the Empty control. Average of biological replicates and SEM are shown. Statistical significance was calculated by two-way ANOVA on ΔΔCt values comparing to Empty vector (*, **, *** = p value < 0.05, 0.01, 0.001 respectively, corrected for multiple testing with Sidak method). **(B)** Examples of gating set ups for cell sorting of SSEA1 and EPCAM1 markers at day 10 of reprogramming upon T7-TIA1 overexpression. Numbers within the rectangles indicate percentages of cells in each gate. **(C)** Barplot representing the percentage of alive ($DAPI^-$, light gray) and dead ($DAPI^+$, dark gray) cells. Average and SD of days 6, 8, 10 and 12 upon T7-TIA1 overexpression are shown for each condition. Statistical significance was calculated by Fisher's exact test against Empty condition (*, **, *** = p value < 0.05, 0.01, 0.001 respectively). **(D)** Gene expression of *Lef1* gene (cpm values, blue line, right y axis) and PSI values of *Lef1* exon 6 and 11 (grey lines, left y axis) in B cell reprogramming. **(E)** Gene expression of *Lef1* gene (cpm values, magenta line, right y axis) and PSI values of *Lef1* exon 6 and 11 (grey lines, left y axis) in MEF reprogramming. Asterisks point out PSI values calculated with low coverage (less than 10 actual reads). **(F)** *Lef1* pre-mRNA isoforms including exons 6 and 11 (*Lef1*-inclusion) or skipping both (*Lef1*-skipping) are represented. Thin lines represent introns, while wider regions represent exons. Lighter and thinner parts of exons depict 5' and 3'UTR regions. Alternative exons are highlighted. **(G)** Relative expression levels of *T7-Lef1* mRNA quantified by RT-qPCR using specific primers that only amplify exogenous T7-tagged *Lef1*. The y axis represents relative expression (2^(-ΔCt) value) of *T7-Lef1* after normalization over *Gapdh*. **(H)** Relative expression levels of *Pou5f1* and *Nanog* quantified by RT-qPCR as in panel D. (G,H) Average of biological replicates and SD values are shown (n=4). Statistical significance was calculated by *t*-test on ΔCt values comparing to the Empty control (*, **, *** = p value < 0.05, 0.01, 0.001 respectively, corrected for multiple testing with Holm-Sidak method). **(I)** Increase in early reprogramming intermediates at day 6 post-OSKM induction upon overexpression of *Lef1* isoforms. Fold change was calculated from the percentage of SSEA1+EPCAM1- cells (of the total of alive cells) in every condition compared to Empty control using flow cytometry analysis. **(J)** Increase in late reprogramming intermediates at days 10 and 12 post-OSKM induction upon overexpression of *Lef1* isoforms. Fold change was calculated from the percentage of SSEA1+EPCAM1+ cells (of the total of alive cells) in every condition compared to the Empty control using flow cytometry analysis. **(K)** Number of colonies stained with alkaline phosphatase (AP) at day 14 post-OSKM induction upon *Tia1* overexpression. The image of a representative well is shown for every condition. (I,J,K) Average of biological replicates and SD values are shown (n=4). Statistical significance was calculated by *t*-test comparing to the Empty control (*, **, *** = p value < 0.05, 0.01, 0.001 respectively, corrected for multiple testing with Holm-Sidak method).

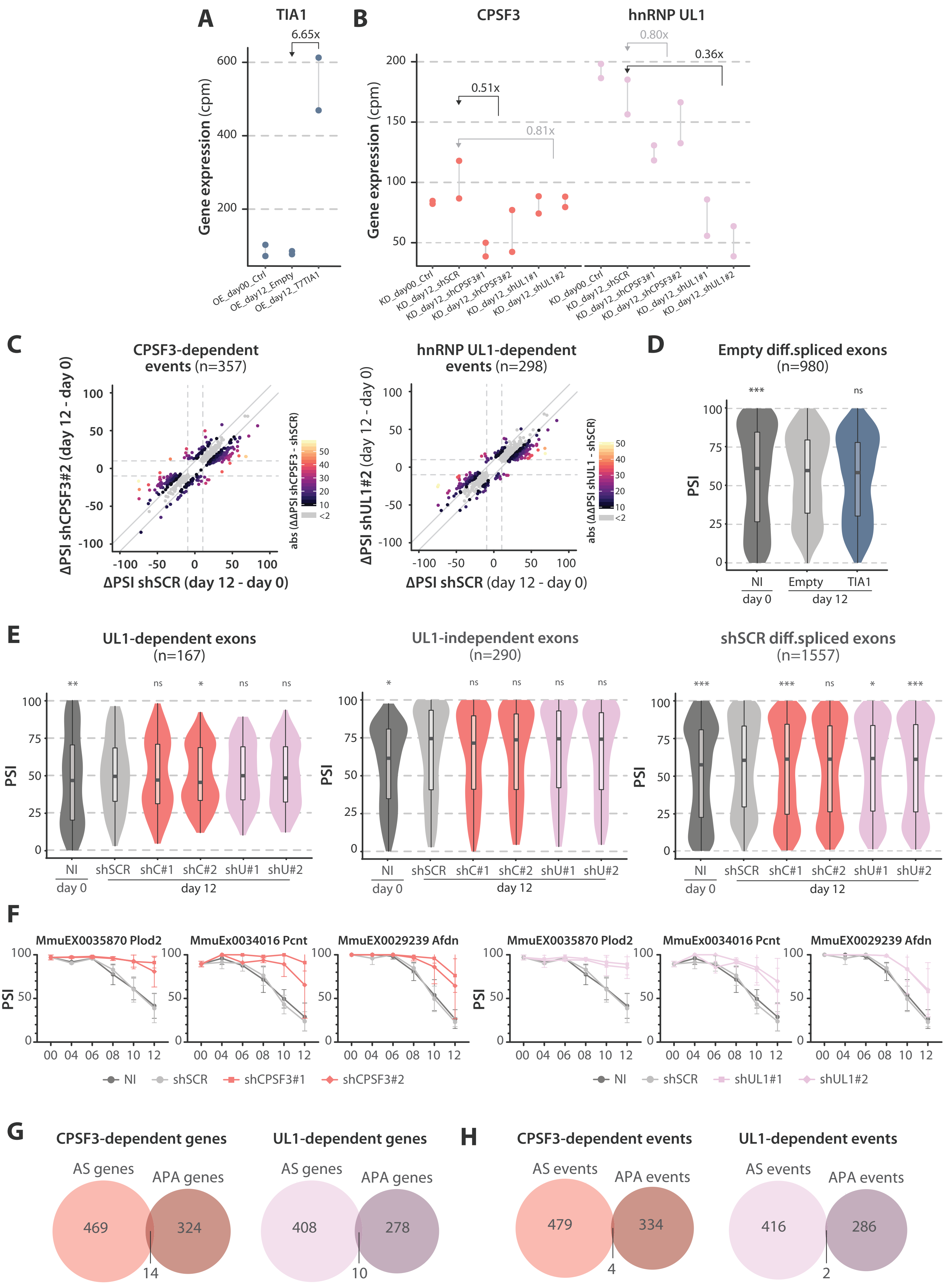

Figure S6 *(legend on next page)*

Figure S6. (A) Expression levels (cpm values) of *Tia1* in the corresponding RNA-seq dataset. Fold change between cells overexpressing *T7-Tia1* and control cells transduced with an Empty vector is shown (day12, average between replicates). **(B)** Expression levels (cpm values) of *Cpsf3* and *Hnrnpul1* in the corresponding RNA-seq dataset. Fold change values between cells infected with specific shRNAs and control shSCR are shown (day12, average between replicates and shRNAs). **(C)** CPSF3- and UL1-dependent events detected during reprogramming (left and right, respectively), representing the ΔPSI with the second shRNA (as in Figure 6C). The x axis represents the ΔPSI value between shSCR day 12 and day 0 control. The y axis represents the ΔPSI value between shCPSF3#2 or shUL1#2 day 12 and day 0 control. Only the events differentially spliced with both CPSF3- or hnRNPUL1-specific shRNAs are shown. Events coloured with the palette indicated are defined as TIA1-dependent events (average |ΔΔPSI(shRNA - shSCR)| ≥ 10), while the others are represented by grey dots. **(D)** Relative to Figures 6E-F. Violin plots representing the distribution of PSI values of exons differentially spliced during reprogramming in Empty conditions (|ΔPSI(Empty day12 – NI day 0)| ≥ 10 and range ≥ 5). **(E)** Relative to Figures 6J-K. Violin plots representing the distribution of PSI values of UL1-dependent/-independent exons (top and central panel) and exons differentially spliced during reprogramming in shSCR conditions (|ΔPSI(shSCR day12 – NI day 0)| ≥ 10 and range ≥ 5, bottom panel). (D,E) Statistical significance was calculated by Fisher's exact test comparing number of events with intermediate (25 < PSI < 75) or extreme PSI values (PSI ≥ 75 or ≤ 25) in each condition against shSCR control (*, **, *** = p value < 0.05, 0.01, 0.001 respectively). **(F)** Inclusion levels of selected CPSF3- and UL1-dependent AS events, quantified by semi-quantitative RT-PCR and capillary electrophoresis. Values represent average and SD. **(G)** Venn diagrams representing the overlap of genes containing CPSF3- and UL1-dependent AS events (lighter color) or APA events (darker color). **(H)** Venn diagrams representing the overlap between coordinates of CPSF3- and UL1-dependent AS events (lighter color) and APA events (darker color).

References

1. Cieply B, Park JW, Nakauka-Ddamba A, Bebee TW, Guo Y, Shang X, et al. Multiphasic and Dynamic Changes in Alternative Splicing during Induction of Pluripotency Are Coordinated by Numerous RNA-Binding Proteins. Cell Rep [Internet]. 2016 Apr;15(2):247–55. Available from: https://linkinghub.elsevier.com/retrieve/pii/S2211124716302819
